# Is Evidence-Based Medicine Relevant to the Developing World?

**DOI:** 10.1371/journal.pmed.0020107

**Published:** 2005-05-31

**Authors:** Paul Chinnock, Nandi Siegfried, Mike Clarke

## Abstract

Systematic reviews have yet to achieve their potential as a resource for practitioners in developing countries, argue Chinnock et al.

Although there is still some resistance to the evidence-based medicine movement, evidence-based health care has now become widely accepted and adopted. Systematic reviews of the effectiveness of health care interventions are the engine room of evidence-based health care; much has been written about how these reviews should be conducted and what they can achieve [1,2]. If the case for the use of systematic reviews is good in developed countries—and we think it is—then the case is even stronger in the developing world. Wherever health care is provided and used, it is essential to know which interventions work, which do not work, and which are likely to be harmful. This is especially important in situations where health problems are severe and the scarcity of resources makes it vital that they are not wasted [3].

But are the systematic reviews that have so far been published relevant and of practical use to those who provide health care in “the majority world” (i.e., in developing countries? In our view, the relevance of systematic reviews to frontline health care workers in developing countries has so far been limited, for a number of reasons.

## Reasons Why the Relevance Is Limited

### Conditions.

Most of the reviews produced to date address health conditions that are priorities in the developed world [4]. Many major health concerns in developing nations have yet to be made the subject of a review, although there are signs that this may be changing [5]. The introductory discussions of most reviews focus on the impact of conditions in the United States and Western Europe. This may be an indication of the authors' own priorities and experience, or it may be because they have made assumptions about the priorities of journal editors and readers. [Fig pmed-0020107-g001]

**Figure pmed-0020107-g001:**
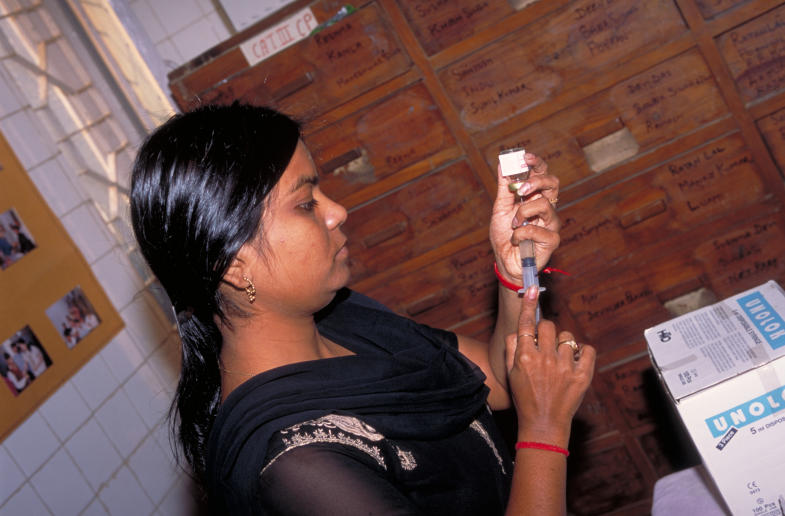
Health care practitioners in developing countries need the most appropriate evidence to guide their practice (Photo: World Health Organization/P. Virot)

### Interventions.

Health care professionals in developing countries sometimes wonder whether their reliance on older, cheaper, “lower-tech” approaches has made their practice quite distinct from that of their colleagues in richer regions [6]. Yet the authors of systematic reviews seem, by and large, to prefer to take on the task of assessing the evidence for more recent (and generally more expensive) technologies. This is not to say that reviewers should avoid high-tech interventions. Again, it is a question of setting priorities, and of recognising the urgent need for more reviews on interventions that are feasible in the majority world.

### Exclusion of studies from the developing world.

Systematic reviews are based largely on research that has been done in rich countries. One of the reasons for this is the relative lack of research in developing countries. However, even when research has been conducted in these countries, it might not be published [7]—or if it is published, it might not be in a journal that is indexed in the widely used bibliographic databases such as MEDLINE and EMBASE. Thus, despite the best efforts of many reviewers, relevant studies may easily be missed. Excluding studies on the basis of language or region is generally not considered good practice in systematic reviewing [8], but the difficulties of identifying and assessing such studies can make finding them and including them in a review an unrealistic expectation.

### Quality of studies from the developing world.

Once studies have been found, they are assessed for quality by the reviewers. Only when the quality meets the criteria specified in the review protocol (in most cases, this specifies randomised controlled trials only) are they included in the analysis. The difficulties of conducting randomized controlled trials in resource-poor situations result in the exclusion of many developing country studies. Some have suggested that the “quality threshold” should be lowered, so that more studies from developing countries can be included in systematic reviews. This question is contentious, and indeed divides the authors of this essay, but it needs to be recognised and debated openly.

### Transferability.

Practitioners in low-income countries have questioned the “transferability” of evidence derived from studies conducted in richer nations [9]. The basis of their concern is their awareness that there can be many differences between patient populations and in the delivery of health care. Forjuoh et al. have pointed out that some injury prevention interventions will have broad transferability, while others will not [10]. They went on to make suggestions as to which intervention would be transferable, but they did so on theoretical grounds without any supporting data.

Features of the typical health care experience of a patient living in the developing world, as compared with features of the typical health care experience of a patient in a clinical trial in a developed country, are shown in Box 1.

**Box 1. Comparison of the Health Care Experiences of Patients in the Less Developed and Developed Worlds**

Features of the typical health care experience of a patient living in the less developed world include

- late presentation
- self-medication of “prescription” drugs or traditional treatments
- poor facilities may delay diagnosis
- referral (if needed) not easily arranged
- if a child, may be malnourished
- if a woman, may be anaemic
- will experience problems because of shortages of trained staff
- …and because of poor infection control
- …and because of a lack of follow-up care
- patient may be unable (e.g., because of lack of funds) to fully adhere to treatment.

Features of the typical health care experience of a patient in a clinical trial in a developed country include

- none of the above

There are also important differences in the way in which care is delivered in developing and developed countries. In developing countries, treatments that would be delivered by doctors elsewhere are often delivered by medical assistants or clinical officers. This may or may not have an impact on the effectiveness of the treatment. Similarly, legislation can be considered a health care intervention for the prevention of road traffic injury, but the “delivery” of such legislation (i.e., its enforcement) is often harder to achieve in developing countries for a multitude of reasons.

As a result of such differences, the most effective treatment in a randomised controlled trial may not be the most effective treatment when provided in the developing world. Some treatments will retain much of their effectiveness in a resource-poor context; others will not.

One recently updated Cochrane review on the primary repair of penetrating colon injuries is a case in point [11]. The update involved the addition of data from one study, which had been completed since the original version of the review had been published. This addition introduced a much greater level of heterogeneity. The likely explanation for this, in the opinion of the reviewers, was that the new study was the only one in which the intervention had been applied in a developing country, which had imposed a number of limitations on its delivery.

Rather than implying that a review's conclusions are globally applicable, perhaps this is one of those circumstances where it would be more appropriate if reviewers concluded with statements such as, “There is evidence for the effectiveness of this intervention in the countries and setting where the included studies were conducted, and in places that are similar in terms of the resources available.”

## What Can Be Done?

It is, of course, vital that more research of quality and relevance is conducted in developing countries, but the writers of systematic reviewers also have much to do. We need to find ways to make a good product better, and we must do more to make sure that people in the majority world are able to access the reviews that are published. In order for progress to be made, the following questions require more attention than they have received up to now.

### Authors.

How can we involve more people from developing countries in the writing and peer reviewing of systematic reviews? For example, how can we continue to build on progress made on international activity within the Cochrane Collaboration [12] (see Table 1)?

**Table 1:**
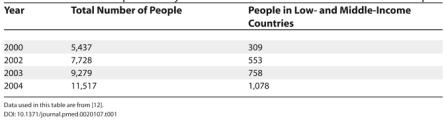
Number of People Actively Involved in Cochrane Collaborative Review Groups

Data used in this table are from [12].

### Titles.

How can we get more reviews written on (a) health problems that are priorities, and (b) interventions that are affordable and feasible in the majority world?

### Context.

Should reviews focus on specific contexts in relation to the location of the condition and the delivery of the intervention?

### Background sections.

How can we encourage reviewers to look at conditions/interventions globally, and not just as they affect the United States and Western Europe?

### Search for studies.

How can we make it easier to find and review data from research done in developing countries?

### Analysis.

Should reviewers be encouraged to consider whether heterogeneity between study results might be due to differences in underlying resources?

### Conclusions.

Should conclusions address whether any recommendations apply everywhere, or just in settings similar to those in which the included studies were done? Or is this beyond the recommendations of a review?

### Dissemination of the findings of reviews.

Is this best done by circulating the reviews themselves, or are reviews merely a stage in the production of more accessible evidence-based health information materials? For example, the World Health Organization's Reproductive Health Library, available on CD-ROM, includes selected Cochrane reviews but also summaries and commentaries that have been specially prepared to provide a developing world perspective. The BMJ's Clinical Evidence produces other summaries of the evidence (for example, often integrating the findings of Cochrane Reviews into answers to clinical questions), and aims to prepare these in user-friendly formats and languages. Are more initiatives like these needed?

### Research.

Research is needed on the impact of systematic reviews on practice in the developing world. We need to assess: What proportion of reviews are relevant to health care in low-resource settings? Are evidence-based sources used to set policy in different countries? How widely are the Cochrane Library and/or Cochrane reviews used by health care workers, and what are the barriers to use? How widely are these resources used by other people involved in decisions about health care, including patients, their carers, and policy makers? Has the use of Cochrane evidence influenced practice? What do these users and potential users think would make reviews more useful?

## Conclusion

When so-called developing countries first gained freedom from their colonial oppressors, Ernst Schumacher pointed out that there was a need, not for the “best” technology, but for “appropriate” technology [13]. When it comes to health care, practitioners and patients of these countries need and deserve nothing less than the most “appropriate evidence”.
